# Determinant Factors on Differences in Survival for Gastric Cancer Between the United States and Japan Using Nationwide Databases

**DOI:** 10.2188/jea.JE20190351

**Published:** 2021-04-05

**Authors:** Yuri Ito, Isao Miyashiro, Takashi Ishikawa, Kohei Akazawa, Keisuke Fukui, Hitoshi Katai, Souya Nunobe, Ichiro Oda, Yoh Isobe, Shunichi Tsujitani, Hiroyuki Ono, Satoshi Tanabe, Takeo Fukagawa, Satoshi Suzuki, Yoshihiro Kakeji, Mitsuru Sasako, Anton Bilchik, Manabu Fujita

**Affiliations:** 1 Department of Medical Statistics, Research & Development Center, Osaka Medical College, Osaka, Japan; 2 Cancer Control Center, Osaka International Cancer Institute, Osaka, Japan; 3 Department of Medical Informatics, Niigata University Medical and Dental Hospital, Niigata, Japan; 4 Department of Gastric Surgery, National Cancer Center Hospital, Tokyo, Japan; 5 Department of Gastroenterological Surgery, Cancer Institute Ariake Hospital, Tokyo, Japan; 6 Endoscopy Division, National Cancer Center Hospital, Tokyo, Japan; 7 Department of Surgery, National Hospital Organization Tokyo Medical Center, Tokyo, Japan; 8 Department of Gastroenterological Surgery, Tottori University, Tottori, Japan; 9 Endoscopy Division, Shizuoka Cancer Center, Shizuoka, Japan; 10 Department of Advanced Medicine Research and Development Center for New Medical Frontiers, Kitasato University School of Medicine, Tokyo, Japan; 11 Department of Surgery, Teikyo University School of Medicine, Tokyo, Japan; 12 Department of Surgery, Kobe University Graduate School of Medicine, Kobe, Japan; 13 Department of Surgery, Hyogo College of Medicine, Hyogo, Japan; 14 Department of Surgical Oncology, The John Wayne Cancer Institute, Santa Monica, California, United States

**Keywords:** stomach neoplasms, survival, registries, lymph nodes, prognosis

## Abstract

**Background:**

Although the incidence and mortality have decreased, gastric cancer (GC) is still a public health issue globally. An international study reported higher survival in Korea and Japan than other countries, including the United States. We examined the determinant factors of the high survival in Japan compared with the United States.

**Methods:**

We analysed data on 78,648 cases from the nationwide GC registration project, the Japanese Gastric Cancer Association (JGCA), from 2004–2007 and compared them with 16,722 cases from the Surveillance, Epidemiology, and End Results Program (SEER), a United States population-based cancer registry data from 2004–2010. We estimated 5-year relative survival and applied a multivariate excess hazard model to compare the two countries, considering the effect of number of lymph nodes (LNs) examined.

**Results:**

Five-year relative survival in Japan was 81.0%, compared with 45.0% in the United States. After controlling for confounding factors, we still observed significantly higher survival in Japan. Among N2 patients, a higher number of LNs examined showed better survival in both countries. Among N3 patients, the relationship between number of LNs examined and differences in survival between the two countries disappeared.

**Conclusion:**

Although the wide differences in GC survival between Japan and United States can be largely explained by differences in the stage at diagnosis, the number of LNs examined may also help to explain the gaps between two countries, which is related to stage migration.

## INTRODUCTION

Although both incidence and mortality of gastric cancer (GC) are showing decreasing trends globally, gastric cancer is still a major health problem worldwide. According to the world estimate of age-standardized incidence rates by the International Agency for Research on Cancer, GC was the cancer with the fifth highest number of cases in the world in 2012. In East Asian countries, including Japan, the number of GC cases was the second highest in 2012. Conversely, in the United States, GC is less common, the incidence rate per 100,000 is much lower than in Japan, about one fifth in men and one sixth in women.^1^

The international collaborative study for cancer survival using population-based cancer registry showed that the survival of GC was higher in Korea and Japan compared with other countries.^2^^,^^3^ The higher survival of GC patients in Japan warrants further investigation. In this study, we compared the survival of GC patients between Japan and the United States and examined the determinant factors of the differences in survival.

## METHODS

### Data sources

#### Japan

We obtained data from the nationwide GC registration project of the Japanese Gastric Cancer Association (JGCA). The JGCA have been collecting clinical data from GC patients in Japan since 1968. Previously, the JGCA published annual reports and detailed survival analysis of patients diagnosed from 1963 to 1991^4^^,^^5^ and updated detailed survival analysis of patients diagnosed from 2001 to 2007.^6^^–^^8^ Over 200 hospitals submitted data from GC patients to the JGCA registration office annually and data from hospitals that submitted more than 50 cases were included in this analysis.

In Japan, although there are population-based cancer registries, detailed clinical information, such as TNM classification and number of LNs examined/resected, are not included. Therefore, the JGCA registry data was used, which includes more specific clinical variables.

We obtained data on 88,447 GC patients registered from 2004–2007. We excluded 9,799 cases: non-surgery or non-resected cases (*n* = 4,670), specific histological type (squamous cell carcinoma, carcinoid, gastrointestinal stromal tumors, and others; *n* = 5,019), missing vital status or follow-up time (*n* = 109), and missing age (*n* = 1). Finally, we analyzed 78,648 cases. We lost 11,210 cases (14.3%) to follow-up (follow-up days <1,825 and alive). The nationwide registration program was approved by the Ethics Committee of the JGCA.

#### United States

We also obtained data from 18,995 patients with gastric cancer from United States population-based cancer registry data from the Surveillance, Epidemiology, and End Results Program (SEER). SEER^*^Stat software is used for the analysis of SEER 18 registries data for research use. In this study, we used data on patients who were diagnosed from 2004–2010 from the November 2015 database version.^9^ We excluded 2,273 cases: specific histological types (squamous cell carcinoma, carcinoid, gastrointestinal stromal tumors, and others; *n* = 2,088) and surgery unknown (*n* = 185). Finally, 16,722 cases were analyzed, of which 2,240 (13.4%) were lost to follow-up (follow-up days <1,825 and alive). The dataset were obtained after approval for our signed Research Data Agreement.

### Variables

We used the following variables as candidate prognostic factors to control: sex, age at diagnosis/surgery (<60, 60–74, ≥75 years), histological type of degree of differentiation (Differentiated: papillary adenocarcinoma [pap]; tubular adenocarcinoma, well differentiated [tub1]; and tubular adenocarcinoma, moderately differentiated [tub2] and undifferentiated: poorly differentiated adenocarcinoma, solid type [SOL/por1]; poorly differentiated adenocarcinoma, non-solid type [NON/por2]; signet-ring cell carcinoma [sig]; and mucinous adenocarcinoma [muc]; in addition to Others or Unknown), location of primary cancer site (Upper [ICD-O-T C16.0 and C16.1], Middle [C16.2, C16.5 and C16.6], Lower [C16.3 and C16.4], Entire Stomach [C16.8], and Missing [C16.9]), UICC TNM classification 6^th^ edition, pathological T (T1, T2, T3, T4, and TX), N (N0, N1, N2, N3, and NX), M (M0, M1, and MX), number of LNs examined (0 or missing, 1–15, 16–20, 21–25, 26–30, and ≥31). Although the SEER used the 6^th^ edition of AJCC for TNM classification, it is comparable with the UICC TNM classification 6^th^ edition. The N of TNM classification was re-categorized as a missing value if the number of LNs examined was smaller than the number of positive LNs, or the number of LNs examined was zero. The SEER data includes a lot of missing data in the TNM classification, we coded as to N0 for NX and “Localized” recorded at the summary stage, and M1 for MX and “Distant” (Table 1).

**Table 1.** Basic characteristics of analysed gastric cancer patients by country

		Japan		US		Total	
		*N*	%	*N*	%	*N*	%
Total		78,648	100.0	16,722	100.0	95,370	100.0

Sex	Male	53,728	68.3	10,474	62.6	64,202	67.3
	Female	24,920	31.7	6,248	37.4	31,168	32.7

Age, years	<60	21,122	26.9	4,624	27.7	25,746	27.0
	60–74	37,813	48.1	6,615	39.6	44,428	46.6
	≥75	19,713	25.1	5,483	32.8	25,196	26.4

Year of surgery	2004	15,679	19.9	2,561	15.3	18,240	19.1
	2005	19,406	24.7	2,445	14.6	21,851	22.9
	2006	21,699	27.6	2,424	14.5	24,123	25.3
	2007	21,864	27.8	2,440	14.6	24,304	25.5
	2008	0	0.0	2,334	14.0	2,334	2.4
	2009	0	0.0	2,277	13.6	2,277	2.4
	2010	0	0.0	2,241	13.4	2,241	2.3

Histological type	Differentiated	41,562	52.8	5,019	30.0	46,581	48.8
	Undifferentiated	36,537	46.5	10,694	64.0	47,231	49.5
	Others/Missing	549	0.7	1,009	6.0	1,558	1.6

Location^a^	Upper	17,435	22.2	4,722	28.2	22,157	23.2
	Middle	30,918	39.3	4,132	24.7	35,050	36.8
	Lower	27,379	34.8	5,043	30.2	32,422	34.0
	Entire	2,810	3.6	1,191	7.1	4,001	4.2
	Missing	106	0.1	1,634	9.8	1,740	1.8

pTNM-T	1	39,067	49.7	3,696	22.1	42,763	44.8
	2	21,234	27.0	7,462	44.6	28,696	30.1
	3	15,332	19.5	3,736	22.3	19,068	20.0
	4	2,851	3.6	1,575	9.4	4,426	4.6
	Missing	164	0.2	253	1.5	417	0.4

pTNM-N	0	44,981	57.2	6,607	39.5	51,588	54.1
	1	18,816	23.9	5,126	30.7	23,942	25.1
	2	7,512	9.6	2,273	13.6	9,785	10.3
	3	4,339	5.5	965	5.8	5,304	5.6
	Missing	3,000	3.8	1,751	10.5	4,751	5.0
	(# of exam = 0 or missing or pos>exam)						

pTNM-M	0	71,876	91.4	14,200	84.9	86,076	90.3
	1	6,772	8.6	2,197	13.1	8,969	9.4
	Missing	0	0.0	325	1.9	325	0.3

# of LN examined	0 or Missing	1,145	1.5	971	5.8	2,116	2.2
	1–15	13,452	17.1	8,769	52.4	22,221	23.3
	16–20 (ref)	8,777	11.2	2,455	14.7	11,232	11.8
	21–25	9,779	12.4	1,595	9.5	11,374	11.9
	26–30	9,339	11.9	923	5.5	10,262	10.8
	≥31	36,156	46.0	2,009	12.0	38,165	40.0

LN, lymph nodes; pTNM, Pathological TNM classification (T, tumor; N, nodes; M, metastasis); US, United States.

^a^Location (ICD 10th code): Upper (C16.0, C16.1), Middle (C16.2, C16.5, C16.6), Lower (C16.3, C16.4), Entire (C16.8), Missing (C16.9).

### Statistical analysis

For the survival analysis, we used the relative survival approach to control for background mortality competing with death from cancer.^10^ For the multivariate time-to-event analysis, we applied an excess hazard model, which was based on Poisson regression using piecewise follow-up time to estimate multivariate effect by controlling for competing risk of death from cancer.^11^ Previously, the Cox proportional hazard model had been applied for time-to-event multivariate analysis, but the model cannot deal with the excess hazard component to control competing risk of death from cancer. For this control, we used country-specific lifetables: for the JGCA data, we used the National Japanese lifetable^12^ and for SEER data, we used race-specific lifetables (Black/White/Others) in the United States, which were provided by SEER^*^Stat.

After the descriptive survival analysis and univariate analysis, we did a multivariate analysis using the excess hazard model to determine which factors might explain the differences in survival between Japan and the United States, with particular focus on the effect of the number of LNs examined. To consider the uncertain data for number of LNs and TNM-N category, we analyzed subgroup data, limited to patients with no distant metastases (M0), and excluded cases for which the number of LNs examined was 0 or missing. We used a multivariate analysis, based on the excess hazard model, including the interaction of country and number of LNs examined, to investigate how number of LNs examined affected the wide differences in survival between the two countries. As number of LNs examined was affected by stage at diagnosis, we also applied the multivariate model, including an interaction term according to N classification. We set 16–20 as the reference category for the analysis that focused on the number of LNs examined, because the UICC/AJCC TNM classification recommendation is to examine over 15 LNs for all surgical cases.

All data analysis was performed using the statistical package Stata ver 13.1 (StataCorp, College Station, TX, USA).^13^ Stata command *strs* and *glm* were used to calculate relative survival based on the Ederer II method and the excess hazard model, based on the Poisson regression model using grouped data.^14^ The statistical significance criterion was set at 5%.

## RESULTS

### Difference in characteristics of patients

We observed distinct differences in distribution of TNM classification, location, and histological grade between the two countries. Only 22.1% patients were diagnosed as T1 in the United States, compared with about 49.7% in Japan. Less than 40% of patients were diagnosed as N0 in the United States, compared with 57.2% in Japan. The distribution of number of LNs examined also showed notable differences between the two countries. In Japan, over 31 LNs were examined in 46% of patients compared with 12% of patients in the United States. In over 50% of patients in the United States, only 1 to 15 LNs were examined (Table 1).

### Differences in observed/relative survival and excess hazard of death from GC

Over 80% of surgically treated patients from the JGCA (Japan) dataset survived 5 years after diagnosis, compared with only 45% in the United States using the SEER database. In most categories, patients in Japan had much higher survival rates than those in the United States (Table 2). All the variables we analyzed were statistically significant prognostic factors. After controlling for confounding factors, all variables were still strong prognostic factors (Table 3).

**Table 2.** Five-year overall survival (5OS) and relative survival (5RS) by prognostic factors and country

		Overall survival				Relative survival			
	
		Japan		US		Japan		US	
		5OS (%)	95% CI	5OS (%)	95% CI	5RS (%)	95% CI	5RS (%)	95% CI
Total		72.1	(71.8 to 72.4)	38.4	(37.5 to 39.3)	81.0	(80.6 to 81.3)	45.0	(44.0 to 46.0)

Sex	Male	70.8	(70.4 to 71.2)	37.6	(36.5 to 38.8)	81.2	(80.7 to 81.6)	44.4	(43.0 to 45.7)
	Female	74.8	(74.3 to 75.4)	39.6	(38.2 to 41.1)	80.5	(79.9 to 81.2)	46.1	(44.4 to 47.8)

Age, years	<60	81.2	(80.7 to 81.8)	45.0	(43.3 to 46.8)	83.2	(82.6 to 83.8)	46.4	(44.6 to 48.2)
	60–74	73.5	(73.0 to 74.0)	41.6	(40.2 to 43.0)	80.5	(80.0 to 81.0)	46.3	(44.8 to 47.9)
	≥75	58.5	(57.7 to 59.3)	29.0	(27.5 to 30.4)	79.5	(78.4 to 80.5)	42.4	(40.3 to 44.6)

Histological type	Differentiated	75.8	(75.3 to 76.2)	47.1	(45.5 to 48.6)	87.1	(86.6 to 87.6)	56.7	(54.8 to 58.6)
	Undifferentiated	68.0	(67.4 to 68.5)	34.1	(33.0 to 35.1)	74.2	(73.7 to 74.8)	39.3	(38.1 to 40.5)

Location	Upper	66.2	(65.4 to 66.9)	35.1	(33.6 to 36.7)	74.7	(73.9 to 75.6)	39.8	(38.1 to 41.6)
	Middle	79.9	(79.4 to 80.4)	42.6	(40.9 to 44.3)	88.5	(87.9 to 89.0)	50.2	(48.1 to 52.2)
	Lower	71.6	(71.0 to 72.1)	40.8	(39.2 to 42.3)	81.5	(80.9 to 82.2)	49.1	(47.3 to 51.0)
	Overlapped	24.7	(22.9 to 26.5)	25.7	(22.8 to 28.7)	27.5	(25.5 to 29.5)	30.4	(26.9 to 33.9)
	Missing								

pTNM-T	1	90.6	(90.2 to 90.9)	66.0	(64.1 to 67.8)	101.0	(100.7 to 101.4)	77.9	(75.7 to 80.1)
	2	69.1	(68.4 to 69.7)	37.6	(36.3 to 38.8)	78.6	(77.8 to 79.3)	44.3	(42.8 to 45.9)
	1–2 (ref. for model)	83.0	(82.7 to 83.3)	46.4	(45.3 to 47.5)	93.2	(92.8 to 93.5)	54.8	(53.5 to 56.1)
	3	35.9	(35.1 to 36.7)	21.5	(19.9 to 23.1)	40.3	(39.4 to 41.3)	24.6	(22.8 to 26.4)
	4	24.8	(23.1 to 26.6)	15.1	(12.9 to 17.5)	28.0	(26.0 to 30.0)	17.1	(14.5 to 19.8)

pTNM-N	0	87.8	(87.4 to 88.1)	58.1	(56.7 to 59.5)	98.3	(97.9 to 98.6)	69.2	(67.5 to 70.8)
	1	61.7	(61.0 to 62.4)	31.3	(29.9 to 32.7)	70.1	(69.3 to 70.9)	36.4	(34.8 to 38.1)
	0–1 (ref for model)	80.2	(79.8 to 80.5)	45.6	(44.5 to 46.6)	90.1	(89.7 to 90.5)	53.8	(52.6 to 55.0)
	2	33.6	(32.5 to 34.8)	15.8	(14.2 to 17.6)	37.6	(36.3 to 38.9)	17.8	(15.9 to 19.7)
	3	17.5	(16.3 to 18.7)	8.4	(6.5 to 10.6)	19.2	(17.9 to 20.5)	9.4	(7.3 to 11.8)
	# of exam = 0 or missing or pos>exam								

pTNM-M	0	77.0	(76.7 to 77.3)	41.7	(40.8 to 42.7)	86.5	(86.1 to 86.9)	49.0	(47.9 to 50.1)
	1	13.4	(12.5 to 14.4)	10.4	(8.7 to 12.2)	15.0	(14.0 to 16.1)	11.7	(9.8 to 13.7)

# of LN examined	1–15	68.1	(67.2 to 68.9)	37.5	(36.3 to 38.7)	80.0	(78.9 to 80.9)	44.8	(43.4 to 46.2)
	16–20 (ref)	72.4	(71.4 to 73.3)	39.8	(37.5 to 42.0)	82.8	(81.7 to 83.9)	46.0	(43.4 to 48.5)
	21–25	73.6	(72.6 to 74.5)	36.8	(34.1 to 39.5)	83.0	(81.9 to 84.0)	42.6	(39.5 to 45.7)
	26–30	74.4	(73.5 to 75.3)	38.5	(35.0 to 42.1)	83.2	(82.2 to 84.3)	44.0	(40.0 to 48.1)
	≥31	72.5	(72.0 to 73.0)	42.3	(39.6 to 44.9)	79.8	(79.3 to 80.3)	47.7	(44.7 to 50.6)

CI, confidence interval; LN, lymph nodes; pTNM, Pathological TNM classification (T, tumor; N, nodes; M, metastasis); US, United States.

**Table 3.** Univariate and multivariate analysis using the excess hazard country-specific model

		Univariate model				Multivariate model			
	
		EHR	95% CI		*P*-value	EHR	95% CI		*P*-value
Country	US	1.000				1.000			
	Japan	0.258	0.250	0.267	<0.0001	0.439	0.423	0.456	<0.0001

Sex	Male	1.000				1.000			
	Female	1.049	1.014	1.085	0.0061	0.938	0.908	0.969	<0.0001

Age	<60	1.000				1.000			
	60–74	1.112	1.071	1.155	<0.0001	1.255	1.210	1.301	<0.0001
	≥75	1.438	1.374	1.504	<0.0001	1.721	1.650	1.796	<0.0001

Histological type	Differentiated	1.000				1.000			
	Undifferentiated	2.304	2.219	2.391	<0.0001	1.304	1.259	1.350	<0.0001

Location	Upper	1.000				1.000			
	Middle	0.430	0.411	0.450	<0.0001	0.634	0.608	0.661	<0.0001
	Lower	0.678	0.651	0.706	<0.0001	0.743	0.715	0.773	<0.0001
	Overlapped	3.216	3.055	3.386	<0.0001	0.996	0.944	1.050	0.869

pTNM-T	1–2 (ref. for model)	1.000				1.000			
	3	7.901	7.610	8.202	<0.0001	2.997	2.881	3.118	<0.0001
	4	12.612	11.978	13.279	<0.0001	3.596	3.405	3.798	<0.0001

pTNM-N	0–1 (ref for model)	1.000				1.000			
	2	7.001	6.742	7.270	<0.0001	2.895	2.780	3.015	<0.0001
	3	11.113	10.657	11.588	<0.0001	4.306	4.095	4.529	<0.0001

pTNM-M	0	1.000				1.000			
	1	9.712	9.382	10.053	<0.0001	2.616	2.519	2.716	<0.0001

# of LN examined	1–15	1.441	1.364	1.522	<0.0001	1.449	1.375	1.527	<0.0001
	16–20 (ref)	1.000				1.000			
	21–25	0.872	0.815	0.933	0.0001	0.864	0.812	0.919	<0.0001
	26–30	0.762	0.709	0.818	<0.0001	0.801	0.749	0.856	<0.0001
	≥31	0.819	0.776	0.864	<0.0001	0.706	0.670	0.745	<0.0001

95% CI, 95% Confidence Interval; EHR, excess hazard ratio; LN, lymph nodes; pTNM, Pathological TNM classification (T, tumor; N, nodes; M, metastasis); US, United States.

### Effect of the number of LNs examined

**Figure 1.**
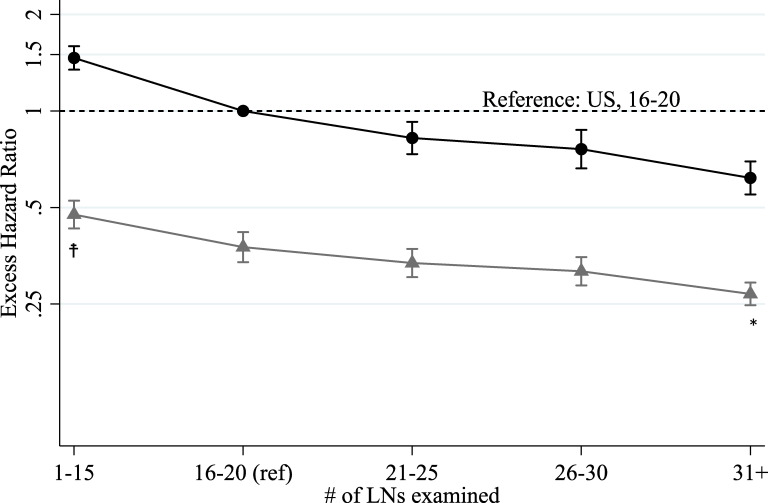
The excess hazard ratios of interaction between number of LN examined and country (Japan); Reference: United States and #LN examined 16–20, including all non-metastasis cases, Black dots: United States and Gray triangles: Japan, 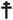 is statistically significantly lower EHR than 1 of the interaction of Japan, and ^*^ is statistically significantly higher EHR than 1 of the interaction of Japan, Black and gray bar lines: 95% CI and statistical significance criterion is 5%. CI, confidence interval; EHR, excess hazard ratio; LN, lymph nodes.

According to the multivariate excess hazard model (Table 3), patients whose number of LNs examined was less (1–15) than UICC/AJCC TNM classification recommendation (≥16), had poorer survival (higher excess death from cancer). Patients whose number of LNs examined was higher than the recommendation showed higher survival. The results from the model for all cases that included the interaction of country (Japan) and number of LNs examined are shown in Figure 1 and eTable 3. The widest gap between two countries was observed in the lowest number of LNs (1–15), while the smallest gap was observed in the category when more than 31 LNs were examined.

**Figure 2.**
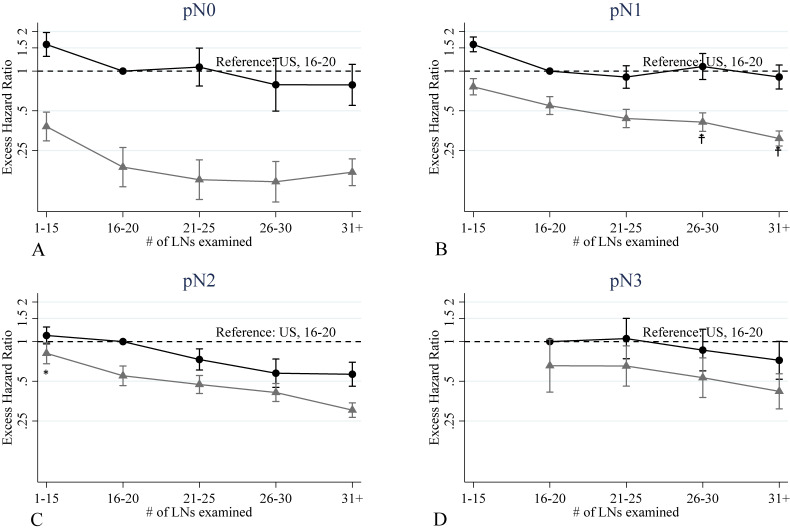
The excess hazard ratios of interaction between number of LN examined and country (Japan); Reference: United States and #LN examined 16–20, results from pathological N-classification specific models, Black dots: United States and Gray triangles: Japan. 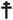 is statistically significantly lower EHR of the interaction of Japan, and ^*^ is statistically significantly higher EHR of the interaction of Japan, Black and gray bar lines: 95% CI and statistical significance criterion is 5%. CI, confidence interval; EHR, excess hazard ratio; LN, lymph nodes.

In the N classification-specific analysis, for N0 patients, regardless of the number of LNs examined, wide differences in excess hazard of death between countries were observed (Figure 2A). For N1 patients, better prognosis was observed patients in Japan in those patients with a higher number of LNs examined number (Figure 2B). For both N0 and N1 patients in the United States, there was no improvement following an increase in the number of LNs examined, while patients in the category 1 to 15 showed worse survival. (Figure 2A and Figure 2B). For N2 patients in both countries, a higher number of LNs examined led to a decrease risk of death. The differences between the two countries were predominantly in the 16–20 and ≥31 categories (Figure 2C). For N3 patients, the differences between the two countries were reduced (Figure 2D).

## DISCUSSION

### Summary of findings

We observed notable differences in GC survival between Japan and the United States. Five-year relative survival in Japan was 79.8%, compared with 40.1% in the United States. The distinct difference can be explained largely by the differences in patient characteristics. However, we still observed a large survival advantage in Japan after controlling for differences in those characteristics using multivariate analysis and stratified analysis. The number of LNs examined was an important factor, but this showed different trends according to TNM-N category. Among N0 and N1 classification patients, higher excess hazard (lower survival) was observed in patients with less than 16 LNs examined than in patients with 16–20 LNs examined in both countries. Among N0 and N1 patients in Japan, much lower excess hazard (higher survival) was observed than in the United States. Also the higher the number of LNs examined, the higher the survival rate in Japan, but not in the United States. For N2 patients, we observed that a higher number of LNs examined was related to better survival in both countries. For N3 patients, the relationship between higher number of LNs examined and excess hazard attenuated.

The lower stage at diagnosis and better survival in Japan compared with the United States might be related to early detection from screening programs for GC in Japan. Population-based GC screening using photofluorography started in the 1980s, under the Health Service Law for the Aged, and other opportunistic screening, such as endoscopy, has also been conducted in workplaces or private medical check-up clinics in Japan.^15^ Although the proportion of participants in GC screening at the local public health service is low, people aged over 40 in Japan have more opportunity for early-stage diagnosis than those in the United States due to greater awareness of the risk of GC and the importance of early detection in a clinical setting. Better survival in Japan is mainly related to the lead-time of early detection due to screening/better awareness.

The distribution of the number of LNs examined also differs between Japan and the United States. Patients in Japan had more LNs examined than in the United States leading to stage migration—the more LNs were examined, the more patients were diagnosed as a later stage. In the less LNs examined group, the diagnosed stage would be earlier, even if the patients were at a more advanced stage. As a result, survival at the same stage of the more examined group was higher than in the less examined group.^16^ According to the stratified multivariate analysis by N classification, for the N0, N1, and N2 patients, there were wide differences in survival between the two countries. However, in the results of N3 patients, the differences between countries were reduced, regardless of the number of LNs examined. For the N2 patients, the more LNs examined, the more favorable the survival in both countries. Although it is difficult to remove bias to compare the two countries, stage migration, related to the more detailed retrieving strategy for LNs in Japan, is a key explanation for high survival in Japan.

### Review of previous studies

A study using combined advanced GC patients’ data from Korea (Yonsei University) and United States (SEER), identified retrieval of 29 LNs as optimal based on the relationship between survival and the number of LNs retrieved. When 29 or fewer LNs were retrieved, it could be seen that the more LNs retrieved, the longer the mean and median survival. However, no improvement in mean and median survival was observed when more than 31 LNs were retrieved.^17^ In our analysis, although a similar trend was observed, we could not analyze the relationship between number of LNs and the prognosis of the patients from whom over 31 LNs were retrieved, because the number of patients in this category was small in the United States database.

### Limitations

We suspect there would be differences in the methods used to examine LNs between two countries, because there were enormous differences in the distribution of the number of LNs examined. Even if the number of LNs examined was the same, the coverage of the area of retrieved LNs might be different between the two countries. In our analysis, we were not able to evaluate this.

As the SEER is a population-based cancer registry, which includes some clinical data, the results are an accurate representation of the United States general population, although it covers less than 30% of United States patients. However, the JGCA data were collected data from some hospitals on a voluntary basis, which may have caused selection bias. In our study data, we could not analyze detailed treatment information, such as surgical procedures and neo-adjuvant chemotherapy and radiation therapy. This may cause confounding in a comparison of survival between the two countries. In the near future, we would like to use population-based cancer registry data linked to clinical databases to deal with this limitation.

In addition, although Lauren’s classification has often been used to categorize histological subgroup in other studies, we did not use it due to difficulties in harmonizing both databases perfectly with the classification. As the subgroup type of differentiation was used in the report from JGCA,^18^ we used the same classification, which can harmonize with SEER data. Although we also applied imperfectly categorized Lauren’s classification, the results did not change.

In conclusion, the wide differences in gastric cancer survival between Japan and United States can be largely explained by differences in patient characteristics, such as the stage at diagnosis. Differences in stage at diagnosis were related to early detection in Japan, mainly due to the screening system and greater awareness of gastric cancer. Stage migration, which is related to differences in the number of retrieved and examined LNs, also helps explain the wide gap in gastric cancer survival between the two countries. Although it is very difficult to remove these biases to compare the two countries in total, for the N2 patients, the more LNs examined, the more favorable the survival in both countries. Compliance with clinical guidelines for diagnosis and treatment could improve gastric cancer care management in both countries.

## APPENDIX A. SUPPLEMENTARY DATA

eTable 1. Basic characteristics of analysed gastric cancer patients by country

eTable 2. Multivariate analysis using the excess hazard model by country

eTable 3. Excess hazard ratios of interaction between number of LNs examined and country based on model for all cases and models by pN classification^a^
