# Supplementary material for: Determinant Factors on Differences in Survival for Gastric Cancer Between the United States and Japan Using Nationwide Databases
Source: J Epidemiol. 2021 Apr 5;31(4):241–8. doi: 10.2188/jea.JE20190351 (PMC7940976; doi:10.2188/jea.JE20190351)
Supplement: Supplementary file 1 [file je-31-241-s001.pdf]

eTable 1. Basic characteristics of analysed gastric cancer patients by country

		Japan		US		Total	
		N	%	N	%	N	%
Total		74,899	100.0	12,476	100.0	87,375	100.0
Sex	Male	51,032	68.1	7,860	63.0	58,892	67.4
	Female	23,867	31.9	4,616	37.0	28,483	32.6
Age, years	<60	20,335	27.1	3,356	26.9	23,691	27.1
	60–74	35,990	48.1	5,069	40.6	41,059	47.0
	≥75	18,574	24.8	4,051	32.5	22,625	25.9
Year of surgery	2004	14,695	19.6	1,831	14.7	16,526	18.9
	2005	18,249	24.4	1,749	14.0	19,998	22.9
	2006	20,763	27.7	1,765	14.1	22,528	25.8
	2007	21,192	28.3	1,852	14.8	23,044	26.4
	2008	0	0.0	1,753	14.1	1,753	2.0
	2009	0	0.0	1,742	14.0	1,742	2.0
	2010	0	0.0	1,784	14.3	1,784	2.0
Histological type	Differentiated	39,772	53.1	4,138	33.2	43,910	50.3
	Undifferentiated	35,127	46.9	8,338	66.8	43,465	49.7
Location	Upper	16,595	22.2	3,957	31.7	20,552	23.5
	Middle	29,532	39.4	3,433	27.5	32,965	37.7
	Lower	26,214	35.0	4,168	33.4	30,382	34.8
	Entire/Overlapped	2,558	3.4	918	7.4	3,476	4.0
pTNM-T	1	37,428	50.0	2,711	21.7	40,139	45.9
	2	20,457	27.3	5,985	48.0	26,442	30.3
	3	14,458	19.3	2,786	22.3	17,244	19.7
	4	2,556	3.4	994	8.0	3,550	4.1
pTNM-N	0	44,645	59.6	5,132	41.1	49,777	57.0
	1	18,538	24.8	4,525	36.3	23,063	26.4
	2	7,415	9.9	1,987	15.9	9,402	10.8
	3	4,301	5.7	832	6.7	5,133	5.9
pTNM-M	0	68,899	92.0	11,137	89.3	80,036	91.6
	1	6,000	8.0	1,339	10.7	7,339	8.4
# of LN examined	1–15	12,851	17.2	6,836	54.8	19,687	22.5
	16–20 (ref)	8,621	11.5	2,060	16.5	10,681	12.2
	21–25	9,642	12.9	1,349	10.8	10,991	12.6
	26–30	9,213	12.3	796	6.4	10,009	11.5
	≥31	34,572	46.2	1,435	11.5	36,007	41.2

CI, confidence interval;LN, lymph nodes; pTNM, Pathological TNM classification (T, tumor; N, nodes; M, metastasis); US, United States.

eTable 2. Multivariate analysis using the excess hazard model by country

		Japan				US			
		EHR	95% CI		p-value	EHR	95% CI		p-value
Sex	Male	1.000				1.000			
	Female	1.034	0.991	1.079	0.1205	0.986	0.933	1.041	0.6044
Age, years	<60	1.000				1.000			
	60–74	1.211	1.156	1.269	<.0001	1.047	0.983	1.115	0.1579
	≥75	1.439	1.357	1.525	<.0001	1.295	1.210	1.387	<.0001
Histological type	Differentiated	1.000				1.000			
	Undifferentiated	2.120	2.027	2.217	<.0001	1.692	1.589	1.801	<.0001
Location	Upper	1.000				1.000			
	Middle	0.427	0.403	0.452	<.0001	0.797	0.744	0.855	<.0001
	Lower	0.706	0.671	0.744	<.0001	0.841	0.788	0.898	<.0001
	Overlapped	4.337	4.077	4.615	<.0001	1.400	1.274	1.539	<.0001
pTNM-T	1–2 (ref. for model)	1.000				1.000			
	3	12.534	11.902	13.199	<.0001	2.319	2.187	2.459	<.0001
	4	19.572	18.287	20.948	<.0001	3.219	2.969	3.490	<.0001
pTNM-N	0–1 (ref for model)	1.000				1.000			
	2	9.303	8.871	9.757	<.0001	2.707	2.544	2.881	<.0001
	3	15.966	15.177	16.795	<.0001	3.812	3.508	4.143	<.0001
pTNM-M	0	1.000				1.000			
	1	13.593	13.044	14.165	<.0001	3.309	3.093	3.539	<.0001
# of LN examined	1–15	1.239	1.142	1.344	<.0001	1.055	0.979	1.136	0.162
	16–20 (ref)	1.000				1.000			
	21–25	0.965	0.882	1.055	0.4305	1.098	0.992	1.215	0.0703
	26–30	0.936	0.855	1.024	0.1501	1.039	0.920	1.174	0.5384
	≥31	1.135	1.057	1.218	0.0005	0.989	0.893	1.095	0.8264

CI, confidence interval; EHR, excess hazard ratio; LN, lymph nodes; pTNM, Pathological TNM classification (T, tumor; N, nodes; M, metastasis); US, United States.

eTable 3. Excess hazard ratios of interaction between number of LNs examined and country based on model for all cases and models by pN classification[a]

		US				Japan[b] (Reference to US 16-20)			Japan[c] (interaction term)			
	# of LN examined	EHR	95% CI		p-value	EHR	95% CI		EHR	95% CI		p-value
All cases	1–15	1.441	1.364	1.522	<.0001	0.475	0.430	0.525	0.863	0.757	0.984	0.028
	16–20	1.000	(ref)			0.376	0.338	0.419	1.000	(ref)		
	21–25	0.872	0.815	0.933	0.0001	0.336	0.303	0.371	1.083	0.927	1.266	0.315
	26–30	0.762	0.709	0.818	<.0001	0.316	0.286	0.350	1.106	0.931	1.315	0.253
	≥31	0.819	0.776	0.864	<.0001	0.269	0.248	0.291	1.156	1.001	1.335	0.048
pN0	1-15	1.594	1.293	1.965	<.0001	0.380	0.296	0.488	1.275	0.874	1.859	0.207
	16-20	1.000	(ref)			0.187	0.133	0.263	1.000	(ref)		
	21-25	1.073	0.771	1.494	0.676	0.150	0.106	0.212	0.748	0.447	1.252	0.269
	26-30	0.788	0.497	1.249	0.311	0.145	0.102	0.206	0.983	0.534	1.810	0.956
	31+	0.786	0.549	1.125	0.189	0.171	0.135	0.216	1.164	0.728	1.862	0.526
pN1	1–15	1.594	1.402	1.812	<.0001	0.761	0.660	0.877	0.873	0.727	1.047	0.143
	16–20	1.000	(ref)			0.547	0.468	0.639	1.000	(ref)		
	21–25	0.902	0.740	1.098	0.303	0.437	0.373	0.513	0.887	0.693	1.134	0.338
	26–30	1.084	0.864	1.359	0.487	0.411	0.349	0.482	0.692	0.528	0.909	0.008
	≥31	0.902	0.732	1.112	0.334	0.308	0.271	0.352	0.625	0.491	0.795	<.0001
pN2	1–15	1.113	0.960	1.289	0.156	0.815	0.679	0.977	1.330	1.045	1.694	0.021
	16–20	1.000	(ref)			0.551	0.464	0.654	1.000	(ref)		
	21–25	0.731	0.608	0.880	0.001	0.473	0.404	0.553	1.174	0.915	1.508	0.208
	26–30	0.576	0.450	0.737	<.0001	0.411	0.351	0.481	1.297	0.962	1.747	0.088
	≥31	0.565	0.458	0.698	<.0001	0.302	0.266	0.343	0.970	0.754	1.249	0.815
pN3	1–15[d]	-	-	-		-	-	-	-	-	-	
	16–20	1.000	(ref)			0.656	0.414	1.041	1.000	(ref)		
	21–25	1.054	0.742	1.499	0.768	0.653	0.460	0.928	0.944	0.556	1.600	0.829
	26–30	0.862	0.600	1.240	0.424	0.533	0.377	0.752	0.941	0.554	1.601	0.824
	≥31	0.721	0.518	1.004	0.053	0.420	0.309	0.570	0.887	0.546	1.440	0.627

CI, confidence interval; EHR, excess hazard ratio; LN, lymph nodes; p-TNM, Pathological TNM classification (T, tumor; N, nodes; M, metastasis); US, United States.

[a] All models were adjusted by sex, age at diagnosis, histological type, location and pTNM.

[b] EHRs were reconstructed by the combination of the coefficients of Japan and the interection terms of # of LNs examined.

[c] EHRs were estimated by the model of the interaction term between Japan and # of LNs examined.

[d] Number of patients was too small, excluded in the model.
